# Potential Use of Hyperoxygenated Solution as a Treatment Strategy for Carbon Monoxide Poisoning

**DOI:** 10.1371/journal.pone.0081779

**Published:** 2013-12-02

**Authors:** Xingxing Sun, Hao Xu, Xiangzhong Meng, Jian Qi, Yuanyuan Cui, Yunqing Li, Hui Zhang, Lixian Xu

**Affiliations:** 1 Department of Anesthesiology, School of Stomatology, the Fourth Military Medical University, Xi’an, China; 2 Department of Anatomy, Histology and Embryology, K.K. Leung Brain Research Centre, the Fourth Military Medical University, Xi'an, China; 3 Department of Anesthesiology, the 323 military hospital of the People's Liberation Army, Xi'an, China; University of Louisville School of Medicine, United States of America

## Abstract

**Aim:**

Carbon monoxide (CO) poisoning can cause permanent damage in tissues that are sensitive to hypoxia. We explored the feasibility and efficacy of using a hyperoxygenated solution (HOS) to treat severe acute CO poisoning in an animal model.

**Methods:**

Male Sprague-Dawley rats were subjected to CO poisoning. The HOS was administered into the femoral vein of these rats through a catheter (10 ml/kg). Carboxyhemoglobin (COHb) and blood gases were used to assess the early damage caused by CO poisoning. S100β was measured to predict the development of late cognitive sequelae of CO. The Morris water maze test was performed to assess cognitive function, and Nissl staining was performed to observe histologic change.

**Results:**

The COHb concentrations rapidly decreased at 5 min after the HOS administration; however, the PaO_2_ and SaO_2_ in rats treated with HOS increased significantly 5 min after the HOS administration. The S100β concentrations, which increased significantly after CO poisoning, increased at a much slower rate in the rats treated with HOS (HOS group) compared with the rats treated with O_2_ inhalation (O_2_ group). The escape latency in the place navigation test was shortened after CO poisoning on days 11-15 and days 26-30, and the swimming time in quadrant 4 in the spatial probe test on days 15 and 30 after CO poisoning was prolonged in the rats treated with HOS injection compared with the rats treated with oxygen inhalation or normal saline injection. The neuronal degeneration in the HOS group was alleviated than that in the CO or O_2_ group.

**Conclusion:**

HOS efficiently alleviates the brain damage in acute CO-poisoned rats and thus may serve as a new way to treat human patients with CO poisoning in clinical practice.

## Introduction

Acute carbon monoxide (CO) poisoning continues to be a serious health problem in China and many other countries. It is a common toxic event that confers signiﬁcant long-term morbidity and is associated with severe delayed neuropathology [1]. Despite the aggressive treatment approaches taken in many industrialized countries, the morbidity and mortality rates from CO poisoning have remained high [2]. In addition, half of those who experience CO poisoning have cognitive sequelae between 3 days and 4 weeks after the event, although some survivors with severe CO exposure do remain asymptomatic [3-5]. Hyperbaric oxygen therapy, which uses pure oxygen to speed and enhance the body’s natural ability to heal, has been broadly used as a medical treatment for CO poisoning [6]. However, due to the nature of the equipment required and the inconvenience of ensuring its availability, hyperbaric oxygen therapy often cannot be practically utilized to save a patient’s life. Moreover, for many patients with CO poisoning, hyperbaric oxygen therapy is contraindicated [7-9]. For example, untreated pneumothorax is an absolute contraindication for hyperbaric oxygen therapy. Furthermore, there are side effects of hyperbaric oxygen therapy, such as painful barotrauma affecting the ears and sinuses, oxygen toxicity seizures, pulmonary edema and hemorrhage, and decompression sickness including pneumothorax and nitrogen emboli, and it is also considered a fire hazard [10-12]. A hyperoxygenated solution (HOS) [13] is a potential medical solution developed by Chinese scientists [14,15]. Using photochemistry techniques, oxygen can be dissolved at high concentrations in commonly used medical solutions, such as a 5% glucose solution, normal saline, or lactated Ringer’s solution, thereby converting these solutions into HOSs. The oxygen partial pressure in these solutions can reach 100-120 kPa, which is ten times higher than that in arterial blood [16]. Some animal experiments have proven that the administration of an HOS can partly attenuate the injuries caused by ischemia and hypoxia [17,18]. HOSs are expected to potentially serve as a novel and effective treatment for acute CO poisoning in human patients. To test this hypothesis, we explored the feasibility and efficacy of HOS treatment for severe acute CO poisoning in a rat model.

## Results

### HOS decreased arterial COHb

**Figure 1:**
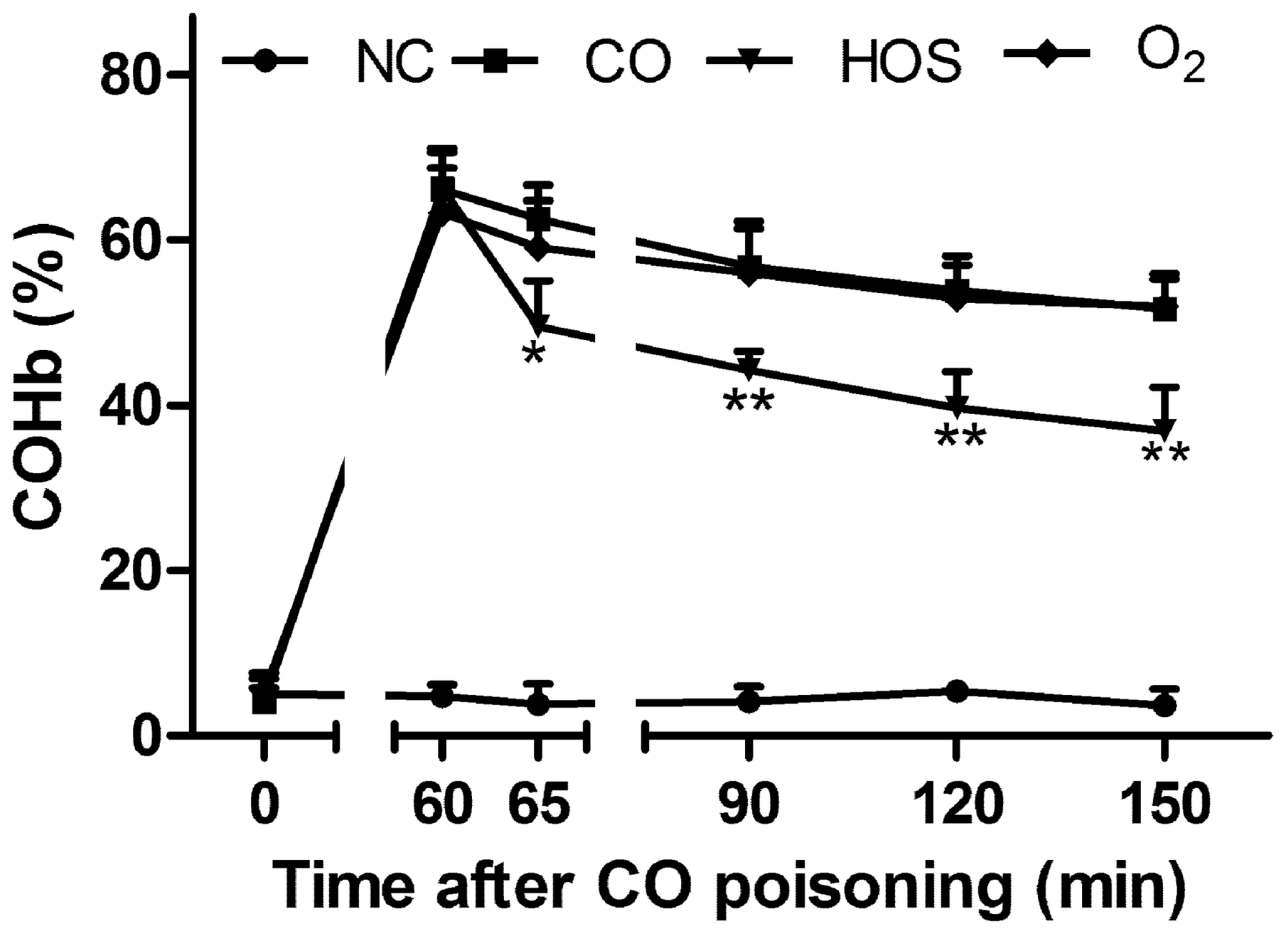
The mean COHb levels at 0, 60, 65, 90, 120, and 150 min after acute CO poisoning. The values are means ± SDs (n = 8). ^*^ comparison of the COHb levels in the CO group with those in the HOS group at each time point (^*^ and ^**^ indicate P < 0.05 and P < 0.01, respectively).

The blood COHb concentrations increased significantly in the CO-poisoned groups compared with the normal control (NC) group (Figure 1). The COHb level peaked approximately 60 min after the CO exposure (64.23 ± 5.30%) and then decreased gradually in all groups. There were no differences in the COHb concentrations between the O_2_ and CO groups at any given time. In contrast, for any given time, the COHb concentrations were significantly lower in the HOS group than in the O_2_ and CO groups. Moreover, the COHb concentrations rapidly decreased 5 min after the HOS administration.

### Effect of HOS on blood gases

**Figure 2:**
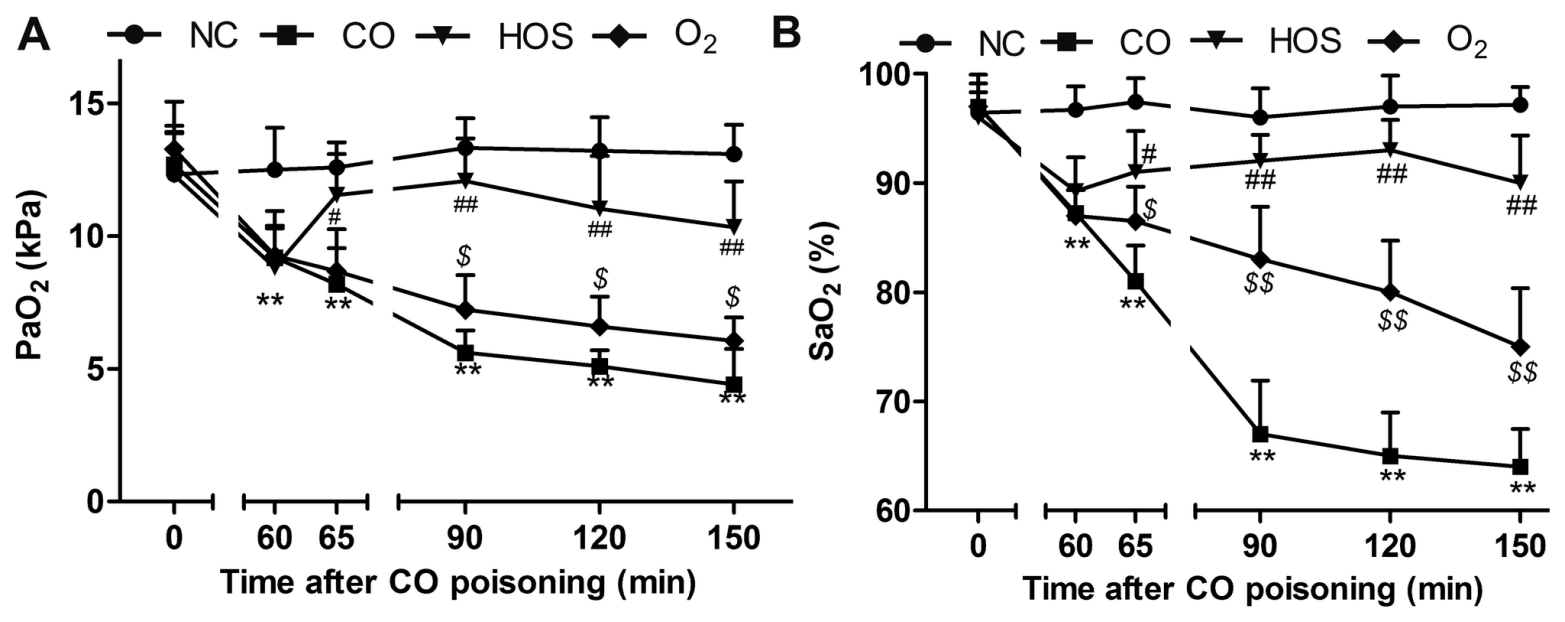
Changes in arterial oxygen partial pressure (PaO_2_) and arterial oxygen saturation (SaO_2_). The mean PaO_2_ levels at 0, 60, 65, 90, 120, and 150 min after acute CO poisoning (A). The mean SaO_2_ level at 0, 60, 65, 90, 120, and 150 min after acute CO poisoning (B). The values are means ± SDs (n = 8).^*^ comparison of the PaO_2_ or SaO_2_ values of the CO group with those of the NC group (^**^ indicates P < 0.01). ***^#^*** comparison of the PaO_2_ or SaO_2_ values of the HOS group with those of the O_2_ group (^#^ and ^##^ indicate P < 0.05 and P < 0.01, respectively). ***^$^*** comparison of the PaO_2_ or SaO_2_ values of the O_2_ group with those of the CO group (^$^ and ^$$^ indicate P < 0.05 and P < 0.01, respectively).

The arterial oxygen partial pressure (PaO_2_) decreased from 12.74 ± 1.63 kPa to 9.09 ± 1.34 kPa, which was considerably lower than that in the NC group (P < 0.01), 60 min after the CO exposure (Figure 2A). Although the PaO_2_ increased significantly in the O_2_ group, it remained consistently lower than that in the NC group. However, the PaO_2_ in the HOS group increased to a normal level 5 min after the HOS was injected into the femoral vein of the rats, which was comparable with that in the NC group (P > 0.05). At 90 min after the CO exposure, although the HOS treatment substantially delayed the PaO_2_ decline, the PaO_2_ in all poisoned groups (CO, HOS, and O_2_) decreased gradually. Similarly, the arterial SaO_2_ decreased to 92.34 ± 2.58%, which was considerably lower than that in the NC group (P < 0.01), 60 min after the CO exposure in all CO-poisoned groups (P < 0.05) (Figure 2B). Although the SaO_2_ was higher in the O_2_ group than in the CO group at any given time (P < 0.01), it decreased progressively even with the O_2_ inhalation. In contrast, HOS injection into the femoral vein of the rats protected against a further decline in the SaO_2_ levels. 

### Effect of HOS on S100β elevation time profile after CO poisoning

**Figure 3:**
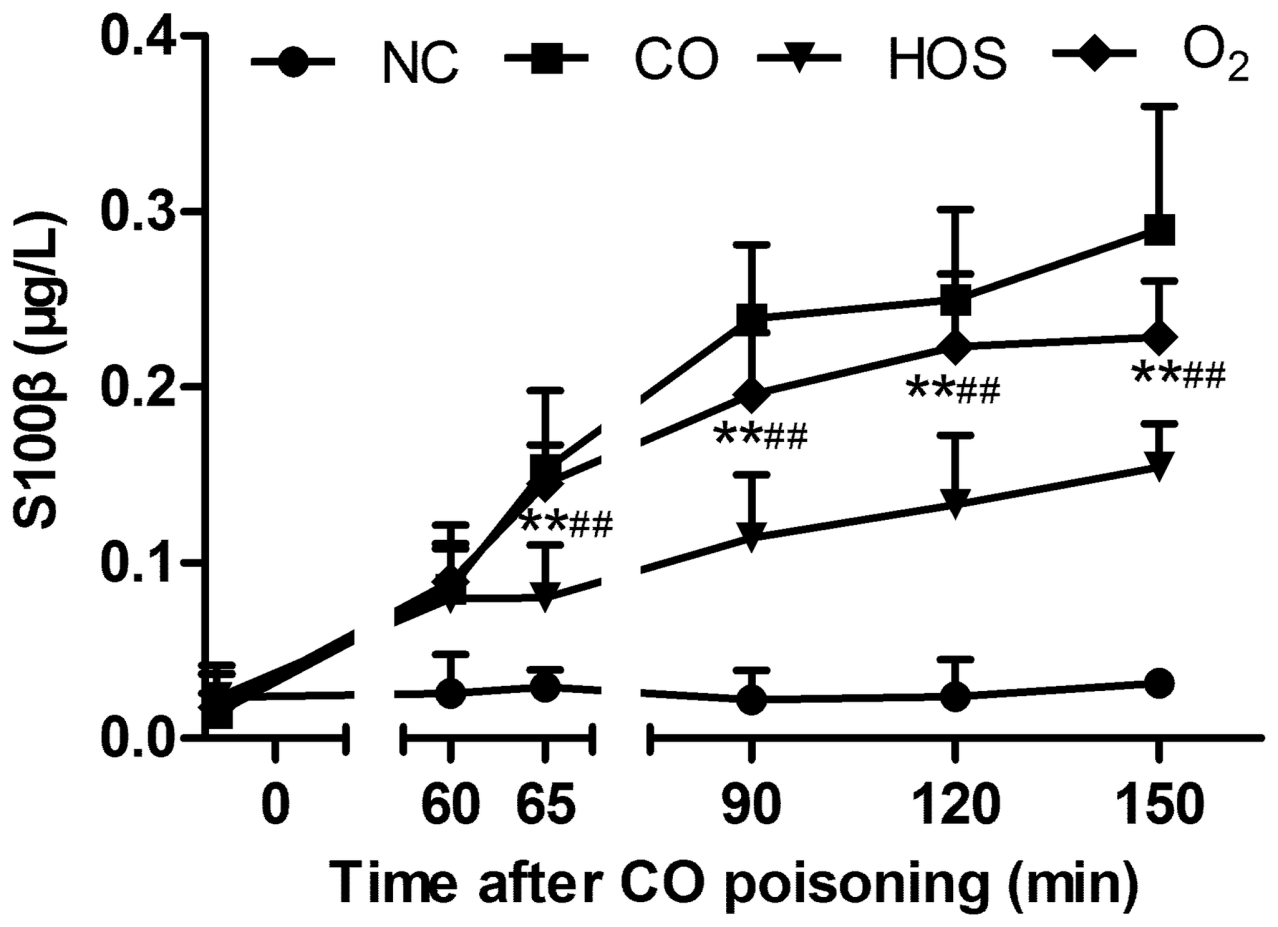
The mean serum S100β levels at 0, 60, 65, 90, 120, and 150 min after acute CO poisoning. The values are means ± SDs (n = 8).^*^ comparison of the serum S100β levels in the HOS group with those in the NC group (^**^ indicates P < 0.01). ***^#^*** comparison of the serum S100β levels in the HOS group with those in the O_2_ group (^##^ indicates P < 0.01).

The serum S100β levels increased significantly in all CO-poisoned groups compared with the NC group (all comparisons P < 0.001, Figure 3), in which the S100β levels remained stable during the experiment (0.02 ± 0.01 μg/l vs. 0.03 ± 0.01 μg/l, P = 0.25). Although the mean levels of S100β in the O_2_ group were lower than those in the CO group at each time point, the differences were not significant. The S100β levels in the HOS group were between those of the NC group and those of the O_2_ group. Moreover, the S100β concentrations in the HOS group increased at a much slower rate than that in the O_2_ group (P < 0.01).

### Spatial learning and memory testing

**Figure 4:**
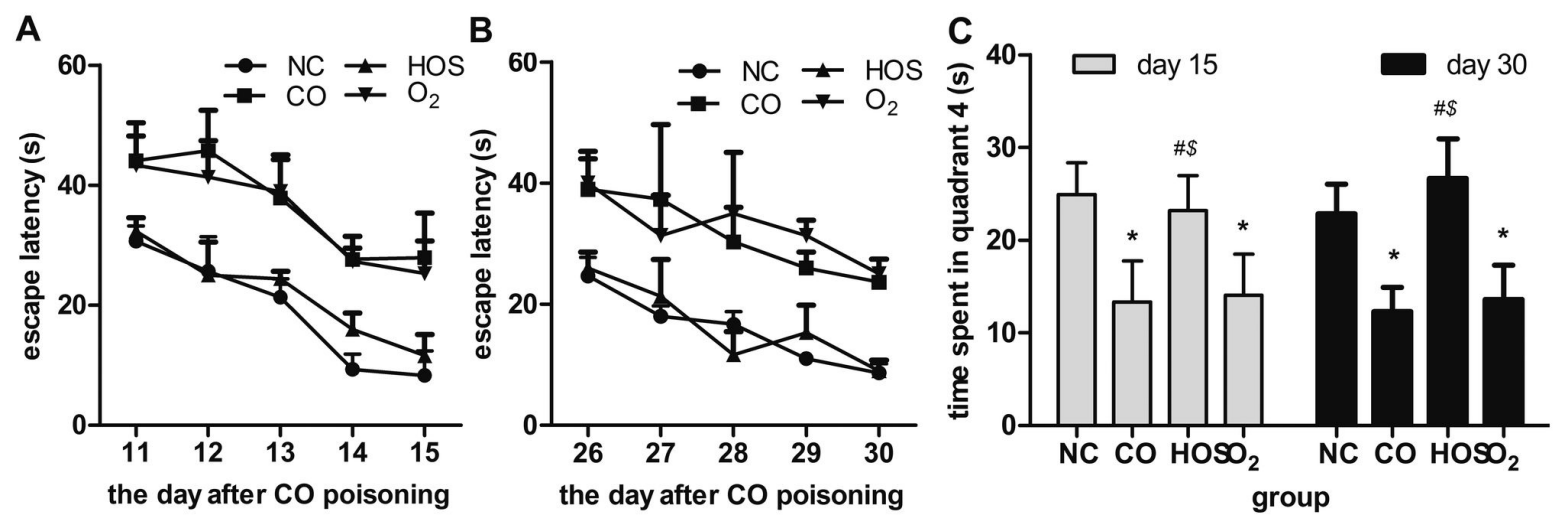
Spatial learning and memory. The means of escape latency on days 11-15 (A) and 26-30 (B) after acute CO poisoning. Swimming time spent in quarter 4 on days 15 and 30 after acute CO poisoning (C). The values are the mean ± SD (n = 3 or 4). ^*^ comparison of the swimming times spent in quarter 4 by the CO or O_2_ group with those by the NC group (^*^ indicates P < 0.05). ***^#^*** comparison of the swimming times spent in quarter 4 by the HOS group with those by the CO group (^#^ indicates P < 0.05). ***^$^*** comparison of the swimming times spent in quarter 4 by the HOS group with those by the O_2_ group (^$^ indicates P < 0.05).

Place navigation test: For each recording day, there was no difference in the escape latency between the O_2_ and CO groups (Figure 4, A and B). A similar pattern was also noted between the HOS and NC groups. The escape latency in the O_2_ and CO groups was significantly longer than that in the HOS and NC groups (all comparisons P < 0.01). Two-way ANOVA (group*days) with repeated measures showed no significant interaction effect (group*days) on both days11-15 (P for interaction = 0.73) and days 26-30 (P for interaction = 0.63).

Spatial probe test: Similarly, there was no difference in the swimming time in quadrant 4 between the O2 and CO groups (Figure 4C). A similar trend was also noted between the HOS and NC groups. The swimming time in quadrant 4 in the O2 and CO groups was significantly shorter than that in the HOS and NC groups (all comparisons P < 0.01).

### Nissl staining

**Figure 5:**
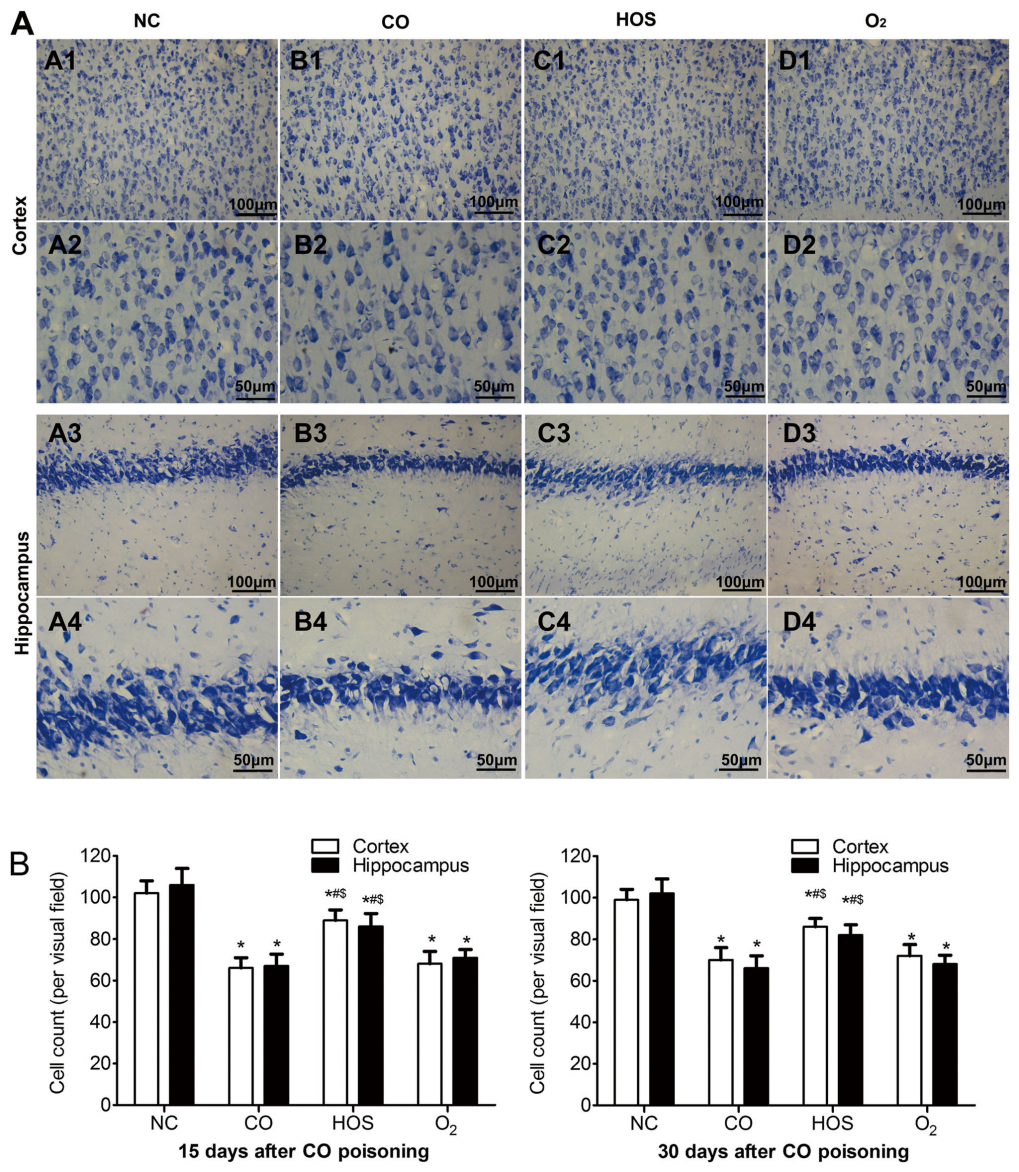
Nissl staining and cell counting. Nissl staining of cortex (A1-D2) and hippocampus (A3-D4) in each group are shown at two different magnifications (A1-D1, A3-D3: ×200, A2-D2, A4-D4: ×400). Cell counts per visual field (×400) found in the slides with Nissl staining on 15 and 30 days after CO poisoning (B). The values are means ± SDs. *P < 0.05 comparing to the NC group; ^#^P < 0.05 comparing to the CO group and ^$^P < 0.05 comparing to the O_2_ group.

Nissl staining was used to illustrate the morphology change of the cortical and hippocampus neurons (Figure 5A). In the NC group, rare injured neuron was detected. The visual field was full of clear and intact neurons, without edema around the cells. However, a significant proportion of neurons in the CO and the O_2_ group were damaged, exhibiting extensive degenerative changes including sparse cell arrangements, loss of integrity, shrunken cytoplasma, oval or triangular nucleus and swollen cell bodies. In contrast, the severity of neuronal degeneration in the HOS group was evidently alleviated than that in the CO or O_2_ group. The number of Nissl staining cells in cortex and hippocampus of CO group was lower than that of NC and HOS groups (P < 0.05), and the number of HOS group was significantly higher than that of O_2_ group (P < 0.05) on both 15 and 30 days after CO poisoning (Figure 5B).

## Discussion

The objective of the current study was to test whether a HOS treatment for acute CO poisoning had protective effects. We found that the injection of an HOS into rats with acute CO poisoning signiﬁcantly delayed or attenuated the increases in the COHb and S100β concentrations and the decreases in the PaO_2_ and SaO_2_ levels. Moreover, the HOS administration considerably enhanced the CO-poisoned rats’ cognitive function, as demonstrated by the place navigation and the spatial probe tests.

The decreasing rate of the COHb concentration is affected by the half-life of COHb, which has been reported to be shortened to 40-80 min and 240-320 min by the treatments with 100% oxygen and room air (21% oxygen), respectively [19]. However, our results showed that rats treated with oxygen inhalation had COHb concentrations similar to those of rats treated with normal saline, indicating that treating rats with oxygen inhalation did not decrease the COHb concentration. A short observation time may partly explain the lack of a difference in the COHb concentrations between the O_2_ and CO groups. Another possible explanation is that spontaneous breathing may be suppressed by CO poisoning. We found that the COHb concentrations started to decrease 5 min after the HOS injection and further decreased to 39.66 ± 4.46% at 90 min, which was much lower than that in the rats treated with oxygen inhalation. This finding suggests that the HOS treatment appears to be effective in attenuating or delaying the damage caused by CO poisoning in the early stages.

PaO_2_ is a good indicator of the amount of physically dissolved O_2_ in the blood. SaO_2_ represents the hemoglobin O_2_ saturation [20]. The PaO_2_ and SaO_2_ improved slightly in the rats treated with oxygen inhalation. In contrast, HOS administration increased the PaO_2_ and SaO_2_ to significantly higher levels compared with general oxygen inhalation, making the body reduce the level of hypoxia more effectively.

HOS has a high oxygen-releasing capacity, the PO_2_ in this HOS was measured to be as high as 100-120 kPa. The oxygen content was 16.28 ± 1.3 mg/ml, two times higher compared to base solution (5.13 ± 0.3 mg/ml). Of note, the HOS we used contained 10-20 mg/l of O_3_ [13,21]. Our prior studies have suggested that the O_3_ could reduce the capacity of red cell aggregation and the viscosity of the blood [22], which would further increase the blood flow velocity and improve the microcirculation in ischemic tissue, thereby reducing the organ damage during hypoxia [23]. Therefore, an HOS infusion that contains a large amount of dissolved O_2_ and a small amount of O_3_ might be an effective way to reduce hypoxia in patients with acute CO poisoning whose hemoglobin cannot deliver enough oxygen to their tissues [24]. In addition, non-cardiogenic pulmonary edema caused by CO poisoning has another important role in the process of tissue hypoxia [25-27]. Our previous study indicated that in addition to hemodynamic amelioration, HOS could improve pulmonary permeability in the lungs of patients and decrease the likelihood of pulmonary edema [17]. Altogether, HOS could effectively relieve hypoxia, which could ultimately prevent or reduce the late cognitive sequelae. 

The late cognitive sequelae of CO poisoning have captured the interest of physicians and researchers. These cognitive sequelae include sensory and/or motor abnormalities, personality changes, and obsessive-compulsive behavior. The S100β protein is a calcium-binding protein produced predominantly by glial cells. Previous studies have shown that the increased S100β protein levels in acute CO-poisoned rats was a useful marker in the assessment of the clinical status and a reliable predictor of neuronal damage and the development of late cognitive sequelae [28-31]. The dysfunction of the blood-brain barrier may result in increased serum S100β levels [32,33]. In our study, we tested S100β in the early stage and found that the HOS treatment could not decrease the S100β level. However, in the very early stage (≤ 150 min) of CO poisoning, the HOS treatment can prevent S100β from increasing to high levels. Although the explanation for this observation remains unclear, the HOS treatment improves the permeability of the blood-brain barrier [34]. We also evaluated cognitive function in the late stage (on the 15th and 30th days) after CO poisoning. We found that a HOS injection attenuated the impairment of cognitive function as evidenced by the findings that the place navigation and spatial probe abilities in the HOS group were better than those in the CO and the O_2_ group and were comparable to those in the NC group. These results support the notion that S100β is a good predictor of the development of late cognitive sequelae.

Our study has several limitations. First, measuring the S100β protein with an immunoassay may produce a false positive result in some rats as a small amount of S100β protein can be found in the fat, skin, and muscle. Second, the limited effect of oxygen inhalation on the cognitive functions may have changed if we prolonged the inhalation time. Third, due to the lack of a relatively long-term evaluation, cognitive sequelae over a longer time are difficult to elucidate.

In summary, the administration of an HOS was effective in delaying or attenuating the increases in the COHb and S100β concentrations and the decreases in the PaO_2_ and SaO_2_ levels. Moreover, the HOS treatment improved the neurobehavioral functions of CO-poisoned rats, as demonstrated by the place navigation and spatial probe tests. Therefore, HOS treatments may serve as a novel treatment for human patients with CO poisoning in clinical practice.

## Materials and Methods

### Chemicals

The HOSs were supplied by Shaanxi Kangle Pharmaceutical Factory in Xian, Shaanxi, China. The procedure and mechanisms of the HOSs were described in our previous study [16]. Pure CO (99.95%) was purchased from Shaanxi Zhida company, xi’an, China. 

### Animals

All animal experiments were performed in accordance with the strict protocols approved by the Institutional Animal Care Committee of the Fourth Military Medical University. Thirty-two adult male Sprague-Dawley rats (220-250 g) were provided by the Experimental Animal Centre of the Fourth Military Medical University, Xi’an, China. All rats were housed in individual cages with free access to food and water and maintained on a 12-h light/dark cycle (08:00-20:00 h) at an ambient temperature of 22 ± 1 °C. The behavior study was conducted from 9:00 to 15:00 h. All efforts were made to minimize the number of animals used and the distress of the animals. The behavior data of the rat (n = 1) in the CO group that died 5 days after the CO poisoning was missing.

### Experiment groups

All rats were randomly assigned to the following four groups: 1) the NC group (n = 8); 2) the acute CO poisoning (CO) group (n = 8); 3) the HOS treatment (HOS) group (n = 8); and 4) the oxygen treatment (O_2_) group (n = 8). 

### Catheter placement

The rats were anesthetized with sodium pentobarbital (50 mg/kg) 2 h before acute CO poisoning. Catheters were inserted in the right femoral artery and vein, and the incision was closed and stabilized with sutures [35]. Heparinized saline (100 U/ml) was used to maintain patency of the catheter.

### Rat model with acute CO poisoning and treatment

The acute CO poisoning model was established according to published protocols [36]. Rats were placed in a self-made plexiglass chamber with a 7-liter volume. Through out the exposure to CO, air was flushed through the chamber at a rate of ~8 liters/min. A CO detector tube in the chamber was used to determine the concentration of the CO gas. The rats breathed 1000 ppm CO gas for 40 min, followed by 3000 ppm CO until they all lost consciousness or 20 min elapsed. Then, all the rats were immediately moved into room air. The non-CO-treated rats in the NC group were placed in the Plexiglas chambers for similar periods of time with exposure to room air. 

After the establishment of the rat model, the HOS group was treated with HOS (10 ml/kg) injection via a catheter in the femoral vein. Both the NC and CO groups received a normal saline (10 ml/kg) injection, and the O_2_ group received pure oxygen inhalation (1 l/min) for 1 h. Each of the treatments in all groups was performed at 0, 24 and 48 h after the establishment of the rat model. 

### Arterial COHb, blood gases

Blood samples (0.2 ml at each time point) were drawn from the right femoral artery of the rats through a catheter at 0, 60, 65, 90, 120, and 150 min after the CO exposure. The blood COHb concentrations were determined using a previously described method, with some modiﬁcations [37]. Each blood sample was diluted approximately 1000-fold in a solution containing Na_2_S_2_O_4_ (2 mg/ml), and the absorbance was measured at 420 and 432 nm using a spectrophotometer (Lambda 35, Perkin-Elmer Corporation, USA). The arterial blood gases were immediately analyzed with a blood-gas analyzer (AVL Compact 3, AVL List, Graz, Austria). 

### S100β testing

Half of the blood samples described above were processed by centrifugation at 3000 rpm for 20 min and then stored at -20 °C until assayed. The serum S100β concentrations were measured with a commercial immune-enzymatic colorimetric assay kit (BioVendor Inc).

### Spatial learning and memory testing

The Morris water maze experiment was performed in a tank with a diameter of 160 cm [38]. The tank was housed in a temperature-controlled room and divided into four quadrants. For each experimental session, the pool was ﬁlled with 20-23 °C water to 30 cm in height. To hide the submerged platform, the water was stained with dark ink. A transparent Plexiglas platform (28 cm in height and 10 cm in diameter) was placed in quadrant 4 (the target quadrant). The platform was submerged approximately 2 cm below the water surface during the learning trials. Three extra-maze cues were set on the wall surrounding the pool. A digital video camera, positioned directly above the pool enabling full collection of the swimming activity in all quadrants, was attached to a computer-controlled system (Jiliang Software Company, Shanghai, China). The animals were initially placed in quadrant 1, 2, or 3 (not containing the platform) with their heads facing the wall. If the animals could not ﬁnd the escape platform within 60 sec, they were gently guided onto the platform by the experimenters and allowed to stay there for 20 sec. For the learning performance, the place navigation test was used. The animals had four 60-sec learning trials daily in which they started in one of four quadrants randomly, with a 20-sec interval between trials. The distance travelled, escape latency to the platform, and time spent in the target quadrant were calculated by averaging the four trial values as indices representing the learning performance. Half of the animals in each group were tested on days 11-15 after receiving the CO or O_2_ treatment, and the other half were tested on days 26-30. Spatial memory was evaluated after the completion of the learning performance tests using the probe test on days 15 and 30; the platform was removed, and the animals were placed in a novel starting position. The time spent in quadrant 4 (platform removed) was calculated as the index for spatial memory. The animals were habituated in the testing room for 30 min before the behavioral tests. After termination of the session, the rat was dried with a towel and placed under a heating lamp before being returned into its home cage.

### Nissl staining and cell counting

After the Morris water maze study on days 15 and 30, the rats were deeply anaesthetized with lethal dose of sodium pentobarbital (50 mg/kg of body weight) and then perfused via transcardial approach with phosphate-buffered saline (50 mL) followed by 4% phosphate-buffered formalin (180 mL). Perfusion-fixed brain tissues were further fixed overnight in the solution (4% paraformaldehyde in phosphate-buffered saline), processed for embedding in paraffin, and cut into 4 μm thick serial sections. For Nissl staining, the sections were hydrated in 1% toluidine blue at 50°C for 20 min. After rinsing with double distilled water, they were dehydrated and mounted with permount. The cortex and the CA1 area of hippocampus from each animal were captured and Imaging-Pro-Plus (LEIKA DMLB) was used to perform quantitative analysis of cell numbers. Six visual fields (0.6mm^2^) of the cerebral cortex and CA1 were photographed in each section. The number of staining cells in each field was counted at higher magnification (×400). The data were represented as the number of cells per high-power field. 

### Statistical analysis

The statistical analysis was performed using SPSS software for Windows, version 16.0 (SPSS Inc. Chicago, IL, USA). Repeated measures ANOVAs were used to compare differences in measures in the treatment groups and time points, with the interactions between factors tested when necessary. One-way ANOVA was used to test for differences among the treatment groups on the separate days in the spatial probe test. The least significant difference *post hoc* test was used when appropriate to compare data among the treatment groups at each time point. For all analyses, P < 0.05 was considered statistically significant. 
